# Embodied niche construction in the hominin lineage: semiotic structure and sustained attention in human embodied cognition

**DOI:** 10.3389/fpsyg.2014.00834

**Published:** 2014-08-01

**Authors:** Aaron J. Stutz

**Affiliations:** ^1^Division of History and Social Sciences, Oxford College of Emory UniversityOxford, GA, USA; ^2^Department of Anthropology, Emory UniversityAtlanta, GA, USA

**Keywords:** embodied cognition, niche construction, hominin adaptation, co-evolution, iconic narrative, semiotics, bipedalism

## Abstract

Human evolution unfolded through a rather distinctive, dynamically constructed ecological niche. The human niche is not only generally terrestrial in habitat, while being flexibly and extensively heterotrophic in food-web connections. It is also defined by semiotically structured and structuring embodied cognitive interfaces, connecting the individual organism with the wider environment. The embodied dimensions of niche-population co-evolution have long involved semiotic system construction, which I hypothesize to be an evolutionarily primitive aspect of learning and higher-level cognitive integration and attention in the great apes and humans alike. A clearly pre-linguistic form of semiotic cognitive structuration is suggested to involve recursively learned and constructed object icons. Higher-level cognitive iconic representation of visually, auditorily, or haptically perceived extrasomatic objects would be learned and evoked through indexical connections to proprioceptive and affective somatic states. Thus, private cognitive signs would be defined, not only by their learned and perceived extrasomatic referents, but also by their associations to iconically represented somatic states. This evolutionary modification of animal associative learning is suggested to be adaptive in ecological niches occupied by long-lived, large-bodied ape species, facilitating memory construction and recall in highly varied foraging and social contexts, while sustaining selective attention during goal-directed behavioral sequences. The embodied niche construction (ENC) hypothesis of human evolution posits that in the early hominin lineage, natural selection further modified the ancestral ape semiotic adaptations, favoring the recursive structuration of concise iconic narratives of embodied interaction with the environment.

## Introduction

Concepts of embodied cognition have been intensively developed and extensively treated in psychology, neuroscience, cognitive science, and the philosophy of cognition over the past 30 years (Humphrey, 1992; Clark, 1993, 1998, 2008; Damasio, 2003, 2008; Gallese and Lakoff, 2005; Rowlands, 2006; Dove, 2011). Although popular accounts have reached scholarly specialists in human evolution (e.g., Coward and Gamble, 2008), embodied cognition seems to be in its conceptual and theoretical infancy in paleoanthropology. Barton's phylogenetic comparative work—focusing on the neuroanatomical support for sensory-motor simulation in the primate order—stands out as a promising exception (Barton, 2012). Paleoanthropology's engagement with embodied cognition research could provide a needed comparative evolutionary perspective on what is unique about how the human body and body-environment interaction shape, facilitate, or constrain cognition.

**Figure 1:**
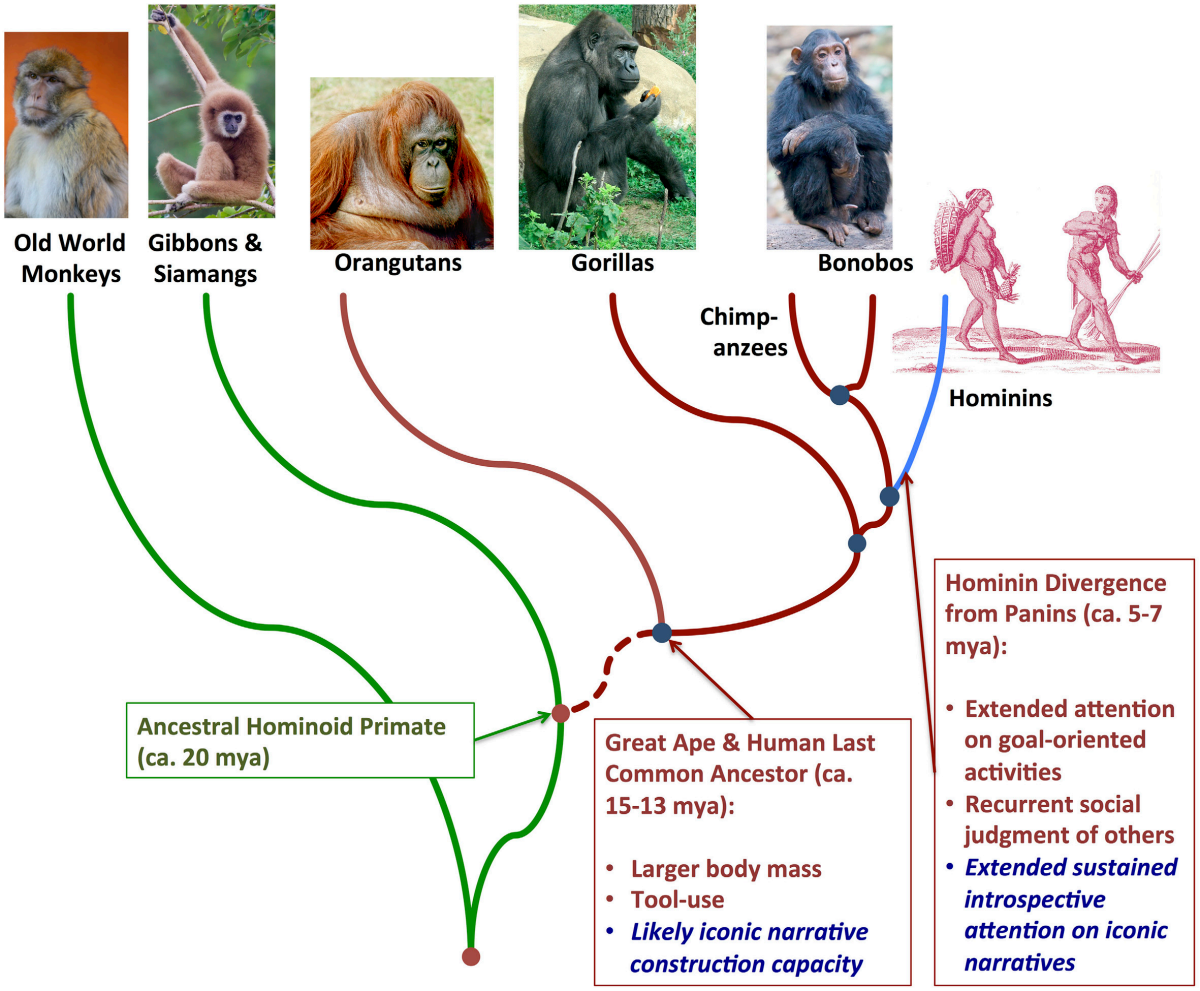
**Phylogenetic relationships among extant anthropoid primate lineages, including Old World monkeys, apes, and humans**. Evolutionarily derived features hypothesized to distinguish embodied niche construction in the nested great ape-hominin and hominin-only clades, respectively, are highlighted in the text boxes lower right.

Such an evolutionary approach highlights some fundamental questions. What aspects of embodied cognition might be relatively evolutionarily primitive among terrestrial vertebrates? What aspects might be relatively derived among the (phylogenetically nested) primate, anthropoid, ape, and hominin lineages, respectively (Figure 1)? I suggest that recent theoretical developments in paleoanthropology, evolutionary biology, and ecology are especially amenable to—and would be scientifically strengthened by—embodied cognition research. Consideration of evolutionary niche construction dynamics (Odling-Smee et al., 2003), in particular, draws our attention to an emerging theoretical intersection, where we can explore how unique human phenotypes—including linguistic communication and symbolic representation—may have co-evolved with a niche significantly constituted by embodied interfaces (1) between the somatic and extrasomatic environments and (2) within the somatic environment itself. The embodied niche construction (ENC) hypothesis aims to complement but achieve more comprehensive explanation of language and sociality than recent proposals in paleoanthropology and linguistics (Deacon, 1998; Jackendoff, 1999; Dunbar, 2003, 2009; Tomasello, 2008). The ENC hypothesis states that human capacities for symbolic mental representation, symbolic communication, and social cooperation emerged over the past ca. 5–7 million years through dynamic co-evolution with embodied cognition and environmental interaction. This occurred within a rather distinctive, very dynamically evolving ecological niche: one that is not only generally terrestrial in habitat—while being flexibly and extensively heterotrophic in food-web connections—but also defined by semiotic, structured and structuring embodied interfaces between the individual organism and the extrasomatic environment.

## Background: toward interdisciplinary common ground

This hypothesis article aims—perhaps naively—to prevail over potential interdisciplinary misunderstanding. I am a biocultural anthropologist (cf. Stutz, 2012) presenting a theoretical speculation about human cognition—and to a specialized cognitive science readership, at that. On the one hand, this is certainly like the student trying to lecture the teacher. On the other hand, it is precisely because I am aware of the importance of cognitive science for studying the embodied dimensions of human experience, awareness, memory, and behavior that I would like to highlight potential areas of common ground. Here, I can make a strong argument that anthropology would benefit from recent theory and research in cognitive science and experimental psychology.

I would further suggest that—for the purpose of interdisciplinary bridge-building (or rebuilding, following mid-late Twentieth Century trends of academic disciplinary proliferation and divergence)—it is worth mapping important anthropological and linguistic terminology and concepts onto relevant psychological and cognitive science ones. For example, in cultural anthropological theory experience and action are widely understood as interrelated, practical, semiotically structured and structuring processes (Sahlins, 2000; Geertz, 2001; Ortner, 2006). There is potentially rich common ground with embodied cognition research, particularly surrounding continuous embodied interaction as a recursive, structured and structuring process that encompasses perception, complex associative learning, episodic memory, simulation and representation.

As with sociological and anthropological theories of practice—where action is always simultaneously symbolically and materially structured and structuring (Bourdieu, 1977; Giddens, 1984; Ortner, 1984)—embodied cognition and continuous environmental interaction is a phenomenon that challenges more than the problematic theoretical distinctions between mind and body. It also blurs distinctions between *representation* and *hierarchically organized, cybernetically regulated action sequences* (Rowlands, 2006; Pfeifer et al., 2007). I would further emphasize that embodied cognition breaks down the apparent boundary between *inter-individual social communication*, on the one hand, and *intra-individual, hierarchically structured feedback through the entire nervous system*, on the other. In fact, it is here that anthropological concepts point toward effectively augmenting recent embodied cognition theories of human language (Clark, 2008; Dove, 2011, 2012). And it is here that anthropology can help cognitive science tackle a key question: if embodied symbol concepts tend to be learned through multimodal sensory-motor experience and remain grounded through evoked sensory-motor simulation, how are abstract concepts successfully constructed and understood (Dove, 2011)?

**Figure 2:**
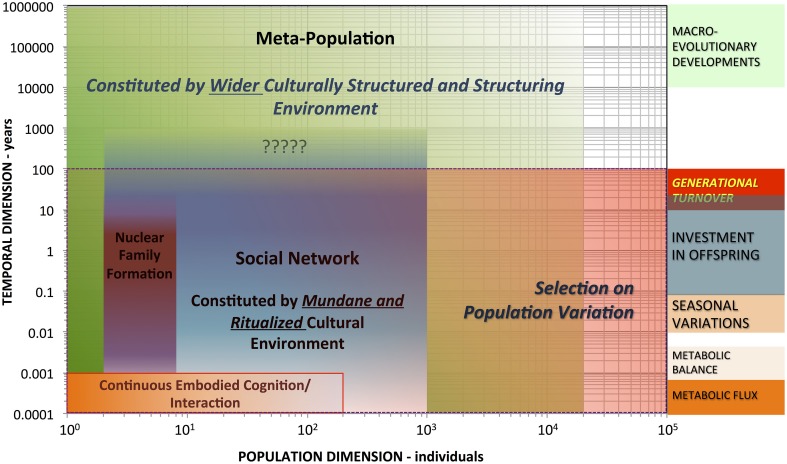
**Logarithmic plot of the general population-time-scale structure of embodied cognition and niche construction in human biocultural evolution**.

With a distinctively anthropological point of departure, the ENC hypothesis can further, more comprehensively guide cognitive science research out of the laboratory, into the wide-open, culturally structured and structuring wild of human cognition (cf. Hutchins, 1996). As Figure 2 schematically illustrates, hominin ENC is proposed to involve non-nested hierarchical system feedbacks from individual, temporally continuous embodied cognition and interaction (lower left) to a metapopulation constituting a cultural environment with elements persisting over millennial timescales (e.g., language structures, technological traditions, and culturally modified landscapes). Ritualized (and ritualizing) cultural environments pervasively structure embodied experience and action. Examples are widely ethnographically and even archaeologically documented. Consider, for instance, how diverse communities handle death's dual crisis—involving both social loss and the emergence of an abject cadaver (Nilsson Stutz, 2003). The cross-cultural diversity in—and patterns of long-term prehistoric and historic change through—mortuary ritual dramatically highlights how human societies construct and practically manage life passages or crises (Nilsson Stutz, 2003). Everyday ritualized handling of embodied experience can also be powerfully transformative, including social interpretation of individual dream experiences—and representations of those experiences (Kracke, 2007). Such qualitative and cross-cultural comparative data support explaining how embodied perceptual representations and concepts could shape and sustain what Dove (2011) has called “dis-embodied” abstract concepts, which emerge from embodied interaction and experience. Abstract but socially useful concepts like being, death, hierarchy, identity, comparison, duty, purity, pollution, and sacred do tend to get materially grounded in embodied social techniques—through dramatic rituals, detailed myths, and production and interaction with artistic objects (Gell, 1998; Nilsson Stutz, 2003). At the same time, these very practices also produce unimaginably rich material for cognitively constructing associations (cf. Clark, 2008; Heyes, 2010a,b, 2012).

In general, the systemic feedbacks among individual memory construction, associative learning, mental planning and social decision-making, on the one hand, and prevailing cultural environments, on the other, combine to shape and constrain how individuals cognize abstract concepts over time, dynamically associating them to the concrete (cf. Lévi-Strauss, 1962a). Still, the embodied cultural environment would seem to be overwhelmingly rich in potentially evocative associations. Thus, the embodied cultural environment would seemingly hinder the individual from sustaining her attention on any one given coherent embodied system of associated extrasomatic environmental stimuli, perceptual experiences, bodily sensations, and remembered experiences. With a prohibitively intricate range of possible, imaginable symbolic boundaries or associative relationships, how is it that we can even construct memory, make behavioral decisions, or achieve enduring attitudes or opinions? This consideration suggests framing the key question another way. If apparently abstract concepts are actually emergent features of complex systems of embodied association production (cf. Clark, 2008), how do such concepts remain robustly connected to a stable association structure? In thought and action, we regularly succeed in making such practical but effectively aesthetic choices (Gell, 1998; Agamben, 1999). I emphasize that theories of embodied cognition—considered from an anthropological perspective—suggest the following prediction. The emotional, proprioceptive, and interoceptive experiences that *result from* successfully constructing or reconstructing socially salient associations between present and past, general and specific, other and self, known and unknown themselves constitute an embodied, unconscious heroic narrative representation of *self successfully constructing a coherent, durable aspect of the world*.

In the remainder of this essay I hope to convince the reader that the ENC hypothesis focuses our own scholarly joint attention on embodied narratives—that is, not simply embodied cognitive simulations, but temporally-compressed, emotionally evocative representations of remembered or imagined events and experiences that would occur over longer time periods, often involving acquisition of durable, even timeless dispositions. Moreover, I hope to make the case that embodied narratives are complex because they are simultaneously adaptive phenotypes and part of our dynamically evolving niche. In this context, embodied narratives have been gradually transformed in the hominin (human) lineage, from private iconic constructions to socially shared, recursively elaborated and endlessly mashed up forms.

## Embodied niche construction: semiotically structured and structuring cognitive interfaces with the environment

When Dawkins described the “long reach of the gene” as its extended phenotype, he argued that DNA replicators drive ecological processes at multiple scales, from intra- to intercellular and from somatic to extrasomatic levels (Dawkins, 1982). The extended-phenotype concept was rhetorically compelling. It was also a heuristic corrective within the history of thought in evolutionary biology. Dawkins argued against overemphasizing or reifying the organism and environment—or even the nature of continuous intergenerational evolutionary change as *constantly* gradual. Yet, this replicator-centered perspective is just as theoretically insufficient for explaining biological evolution as are strictly organism, population, or ecosystem-centered views. Recent work on dynamic niche construction processes (Odling-Smee et al., 2003) and multi-scalar complex ecological processes (Levin, 1992; Gunderson and Holling, 2001; Schneider, 2010) makes clear that biological systems characteristically exhibit resilient structures or equilibrium states at more than one scale, but change at a given scale or structure can have important dynamic feedback effects stretching beyond the local environment. Genetic variation in replicators may drive evolutionary competition and selection, but this is a relatively local ecological complex-systems process. Evolution involves dynamic feedbacks among replicator populations; their non-nested, hierarchically structured extended phenotypes; and their similarly non-nested hierarchically structured niches (Allen and Starr, 1982).

Moreover, there are multiple scales within and around the organism in which phenotypes have a *dual* systemic role. Phenotypes are not just subject to natural selection for fit to the prevailing environment. They are often also the very environments that influence their own fitness (Odling-Smee et al., 2003). In animals ENC occurs because the chordate body is interconnected with the world through complex thresholds, which constitute an integral part of the animal's niche, even as the body is also adaptation to that niche.

It is especially relevant, then, that embodied cognition research emphasizes the dynamic multiscale, often parallel-channel process of an organism's interaction with the environment (Clark, 1998, 2008; Damasio, 2000, 2003; Rowlands, 2006; Pfeifer et al., 2007). In fact, embodied cognition and environmental interaction may be understood as thoroughly intertwined, multidimensional and hierarchically structured processes in which:

- the bodily surface and the sensory organs constitute a complex, interactive threshold between the organism and surrounding environment—in which foraging, predation avoidance, resting, courtship and mating, and other social behaviors unfold; and
- the brain continuously interacts with:
  ➢ the rest of the body—via homeostatic systems that regulate circulatory, metabolic/digestive, motor activity, bodily balance, immune/inflammation, endocrine, reproductive system, and alertness states; and
  ➢ the extrasomatic environment—via sensory organs during joint cognition/bodily action processes; and
  ➢ the subsystems within the brain itself—via:
    – connections among modality-specific perception systems;
    – connections between perceptual representation systems and more specific homeostatic systems, giving rise to feelings and awareness [e.g., metabolic and digestive information increasing alertness, generating an embodied feeling of hunger (cf. Damasio, 2003)]; and
    – simulation (Barsalou, 1999, 2009), memory construction, and other higher-order mental processes that use learned, iconic and indexical neural representations of embodied cognitive/interactive states (cf. Deacon, 1998: Chapter 3), which in turn can affect sustained mental or bodily attention on a cognized object.

With the exception of the simulation and memory construction component, this sketch of embodied cognition/interaction may generally model behavioral processes in vertebrates and most invertebrates. That last component itself—capacity for simulation and learned iconic and indexical embodied representation—does encompass a range of phenotypes found across the vertebrates. In turn, this comparative zoological scope suggests that what Clark (2008:p. 42) has described as “profound” and “promiscuous” embodied engagement with the world is evolutionary quite ancient in the animal kingdom and widespread, especially in the terrestrial biosphere. Finally, as Deacon's (1998: Chapter 3) symbolic-threshold model suggests, the higher-order cognitive capacity to recursively and indexically link symbols, symbols and iconic representations, and deictic symbol-index concatenations could have gradually evolved in the hominin lineage (see Figure 1), with hierarchical complexity increasing over 100's of thousands of years, so that open-ended systems of symbolic representation incrementally pervaded the environments in which embodied interaction occurred.

It is worth pointing out that, here, Chomsky and colleagues' recent general emphasis on symbolic recursion in language (Hauser et al., 2002, 2014) may be seen—at least potentially—as theoretically converging toward Deacon's model, opening the way to a synthetic foundation for investigating the evolution of language as part of a much longer, gradual dynamic, in which embodied cognition and interaction has resulted from a very deep co-evolutionary process. Embodied cognition/environmental interaction has resulted from diversifying niche construction and phenotypic adaptation—via natural selection—across the animal kingdom. I thus suggest that it is particularly important to investigate how embodied cognition/environmental interaction dynamically evolved in the hominins as both niche and phenotype.

### Higher-level cognitive representation in embodied cognitive context

Adaptive interfaces in most vertebrate animal niches constitute—and are constituted by—two non-nested hierarchically structured levels of embodied attention to the immediate surroundings. First, distributed embodied cognitive management of environmental interaction can be monitored and managed through higher-level neural connections in the brain—or even by the relatively distal spinal cord. In either case, environmental interaction effectively proceeds with little centralized higher cognitive interpretation and direction of bodily activity. This allows grasping, gross limb movements, management of torso posture, head movements, chewing, and dynamic gazing/visual scanning to occur efficiently, without constantly raising overall bodily alertness levels—and without overwhelming higher cognitive decision-making systems for determining the present focus of selective attention. Second, higher cognitive processes can take place at the same time as the substantially decentralized orchestration of behavior operates. Higher constructive cognitive processes would be able to manage modal and cross-modal learning and perception of relevant environmental objects, deixis with respect to those objects, memory construction, and—perhaps most importantly—tactical decision-making about changing short-term equilibrium goals.

In evolutionary perspective, this dual-level model of embodied cognition can aid our theorizing about vertebrate animal behavior. For example, it supports our hypothesizing about behaviors of common vertebrate species that thrive in habitats shared with humans. For example, I can reasonably speculate that embodied squirrel (genus *Sciurus*) cognition facilitates sustained manual grasping and rhythmic mastication of a food object, reducing embodied attention on the actual handling and eating behaviors, and thus opening the animal's sensory focus on potential predators, possible mates, and territorial challenges from other squirrels. In general, we may expect that embodied dimensions of cognition have been important in animal evolution, because they may minimize the opportunity costs of selective attention on one fitness determinant—for example, foraging—at the expense of others—including predation risk, conspecific territorial challenges, or courtship and mating.

### Semiotic system construction in embodied cognitive context

The dual-level structure of embodied cognition helps us to investigate the evolution of semiotic construction capacities—initially not for structuring social signaling, but for structuring individual learning, perception, and reasoning. Here, it comes into very sharp focus that “semiotic as logic” is sufficient for learning and memory construction—that is, the cognitive filtering, storage, and recall of relevant information in the animal's ecological context. This is the case, even in the absence of adaptations for complex social communication. Evolutionary primitive cognitive construction of embodied icons and indices is arguably widespread in the mammalian and avian classes. Here, I use Peirce's terminology of signs, following Deacon (1998: Chapter 3), emphasizing that perceived or otherwise cognitively evoked *icons*—as arbitrarily simplified representations of a more complex object—may be dynamically constructed over recurrent embodied interactions with the extrasomatic environment. Moreover, building on the semiotic framework presented in Gell (1998), Peirce's definition of *index* implies that iconic representations also have indexical properties, intrinsically evoking embodied, remembered objects, including other iconic representations. Peirce (2012: Kindle location, 2018) stated, “An Index is a sign which refers to the Object that it denotes by virtue of being really affected by that Object.” The embodied—albeit higher, integrative—cognitive process of icon construction must involve indexical links to perceived objects, which are also deictically, indexically linked to bodily affective states. Thus, icons and their indexical connections form a learned, structured cognitive filter. This functions to convert highly complicated, often noisy extrasomatic and somatic stimuli into consistent, comprehensible body-environment interaction channels. The animal, then, learns to engage in bouts of behavioral activity involving habitual, substantially decentralized embodied cognition and interaction with the environment. At the same time, higher cognitive functions tune attention toward the recurrent abstraction of visual, auditory, or fine, focused haptic perceptions and memories. The resulting pre-linguistic semiotic constructions would thus be mental, private *icons* of more detailed perceptions or simulations. These could include both static imagery and narrative memory. Moreover, these icons would have a secondary semiotic function, *indexically* evoking proprioceptive, interoceptive, and tactile sensations and emotional states, metonymically tied to an iconic representation through prior experience and memory construction. Most basically, I suggest that the evolution of higher cognitive recursive functions that can abstract rich embodied experience and memory into iconic representations likely built on the embodied indexical connection between the following—also dual-level—system of cognition, with the second level further involving embodied indexical links among three particular aspects of cognition:

- visually, auditorily, or haptically mediated construction of extrasomatic objects as alienated from the body
- somatosensory states that affectively represent the condition of part or all of the body, including:
  ➢ interoceptive states
  ➢ proprioceptive states
  ➢ alertness

**Figure 3:**
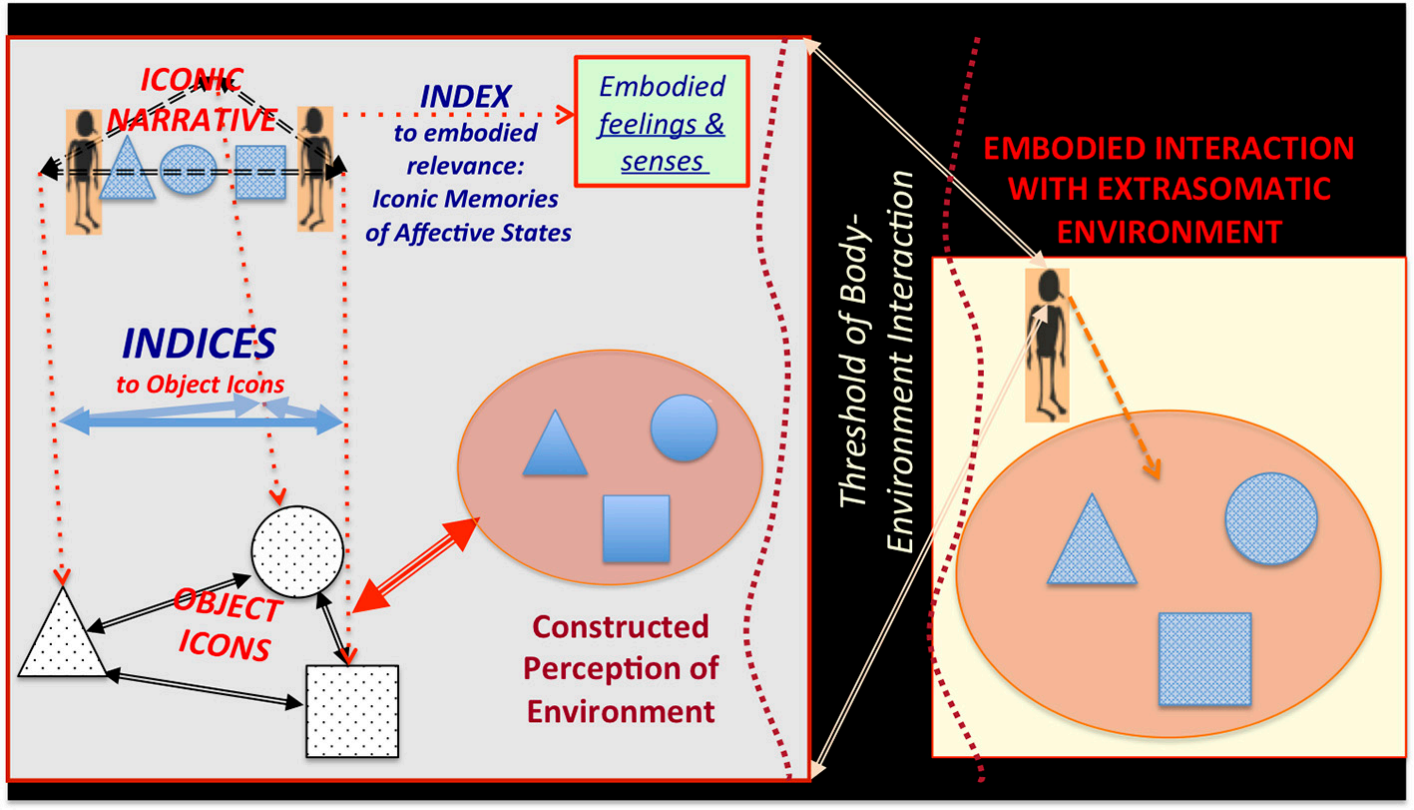
**Hierarchically constructed object icons and iconic narratives—indexically linked to embodied affective states and perceived objects in the extrasomatic environment—via body-environment thresholds**. *Object icons* are sufficiently formed through dynamic, recursive learning. Construction of indexical relationships among icons emerges through embodied interaction with the extrasomatic environment. *Iconic narratives* are constituted by the indexical relationships among object icons and changes in embodied affective, proprioceptive, and interoceptive states. It is hypothesized that one of the most evolutionarily primitive iconic narrative genres—likely evolved in the hominin-great ape common ancestor—is that of heroically succeeding or tragically failing to construct an enduring aspect of the world.

Extending arguments presented in Rowlands (2006), I suggest that—within the context of dynamic experience that structures and is structured by icon-index semiotic systems—we can consider somatic states that are indexically evoked by visually, auditorily, or haptically shaped extrasomatic icons as *embodied icons*. Semiotic, structural linguistic, and anthropological theories of representation have long emphasized that signs are defined not only by the conventionally or logically defined relationship between signifier and signified. They are also defined by their formal relationships to other signs (Jakobson and Halle, 1956; Hockett, 1961; Lévi-Strauss, 1962a,b; Leach, 1976; Saussure, 2011; Peirce, 2012). Following Deacon (1998: Chapter 3), I suggest that the cognitive ability to learn and construct indexical relationships among iconic representations of extrasomatic objects and embodied icons evolved gradually, supporting the recursive construction of concise iconic narrative representations (Figure 3). This would have set the stage for later hominin social manipulation of indexical connections to extrasomatic icons, through gesture and gaze-following (Tomasello, 2008).

In general, the semiotic structuration of embodied cognition is important when partially decentralized cognition and action affords the animal's short-term homeostatic behavior pattern in the extrasomatic environment (Gibson, 1979), freeing up attention toward receiving and decoding information that might imply the *relevance* (cf. Sperber and Wilson, 1995) of altering behavioral and affective homeostatic targets. I speculate that what may have become relatively evolutionarily derived in humans—already early in the divergence of the hominin lineage from that of the panins (see Figure 1)—was the cognitive capacity to construct iconic narratives, in which dramatically changing affective states are temporally contextualized in a representation of a problem (e.g., hunger during the search for food) and its resolution (satiation during feeding). Such iconic narratives should be considered as an emergent part of the embodied interface with the environment, helping the animal to sustain attention on a difficult-to-obtain goal (dragging a stone anvil to the base of a nut tree) or a social dilemma (accepting or rejecting a solicitation to engage in a social coalition). Thus, the recursive nature of constructing icon-index complexes is hypothesized to be an important evolutionary inheritance in hominins, subsequently modified by natural selection to support human symbolic thought and—eventually—communication.

The ENC hypothesis entails that evolution modified the ape “dual-level system” of embodied cognition (see above) in hominin prehistory. Partially decentralized management of locomotion, repetitive tool-making and tool-use gestures, grasping and carrying, and feeding—involving rhythmic or recurrent actions or sustained isometric postures—could be maintained over minutes or hours, while higher cognitive learning, construction, and perception could simultaneously support:

- introspective attention on semiotic signs, their objects, and evoked relationships to other signs
- monitoring of others
- communication, involving solicitation and engagement in bouts of joint attention

I speculate, then, that in early hominins, semiotic representation likely co-evolved with social monitoring and solicitation and sustained engagement in joint attention and interaction, prior to the evolution of spoken language.

### Overview of embodied narratives in hominin niche construction

It is an embodied cognition perspective that makes this possibility apparent. Self's frequent attention on participation in socially intense networks may be punctuated by highly focused, goal-oriented social interactions, technological engagement with the material environment, and ritualized motion sequences. All of these involve reduced alertness, temporarily shutting off embodied interfaces with the wider environment. Thus, managing a marriage alliance, butchering an animal, shaping a wooden digging stick, making a flint hand-axe, or building a hut may involve intense, narrowly selective embodied attention on a multi-step technological process or social interaction, resulting in a material product or negotiated relationship-state. The higher cognitive processes involved in such activities are particularly important for rapid niche construction. The ability to construct abstract, iconic representations from concrete visual, auditory, and haptic perception is an embodied behavioral adaptation. However, the representations themselves—along with their semiotic, indexically or metaphorically evoked connections to other learned signs, perceived objects and events, memories, and embodied mental simulations—become part of the niche, constituting a dynamic part of the interface between the body and the extrasomatic environment (cf. Clark, 2008; Dove, 2011).

The hominin embodied niche is hypothesized to have evolved to encompass a set of dual-level cognitive interfaces with the extrasomatic environment, where rhythmic or sustained static behavior patterns unfold in parallel with complex systems of indexically linked icons, facilitating social observation, action, judgment, and self-awareness. The evolution of bipedal locomotion in the hominin lineage illustrates the complex process of “semiotically constituted and constituting ENC,” examined in the following section.

## A case study: bipedal locomotion as embodied phenotype and niche component

Vertebrate locomotion involves a joint cognitive-behavioral system facilitating the animal's movement through its physical habitat. In general, locomotion itself may be seen as an embodied cognition system, in which control of locomotion is partially—but significantly—decentralized across the central nervous system, peripheral sensory-motor subsystems, and musculo-skeletal subsystems. Central cognitive processing of sensory and other inputs from bodily homeostatic systems is usually minimized, first, through local oscillatory feedback in the limbs, and then through central nervous system management of small homeostatic neuromotor adjustments (Van de Crommert et al., 1998; Dietz, 2003, 2010; Ijspeert, 2008). Finally, the central nervous system supports monitoring a simple series of embodied indices of homeostatic exertion and equilibrium levels of bodily momentum in the immediate extrasomatic environment. These indicators mainly involve the sense of bodily balance, and departures from homeostatic ranges can trigger a cascading increase in local sensory-motor and overall central nervous system alertness, in order to respond to a sudden change in the body's interaction trajectory with the surrounding milieu. We can usually walk—or birds fly—without higher-order cognitive information-processing and decision-making about every heel strike or big-toe push-off—or wing flap. Thus, we can walk, chew gum, play air drums to an imagined tune—and seagulls can scan visually for other members of their flock, prey, and predators—while embodied, distributed cognition takes care of locomotion.

The embodied cognitive niche dimensions of bipedal locomotion are strongly shaped by the fact that, in terms of energy expenditure by the supporting musculo-skeletal and thermoregulatory systems, hominin two-legged walking or jogging uses caloric resources at a substantially lower rate than does great ape quadrupedal walking or running at the same pace (Leonard and Robertson, 1997; Sockol et al., 2007). Efficient, largely decentralized cognitive management of bipedal locomotion synergistically reinforces the biomechanical, energy-saving advantage of bipedal stride or jogging gait in a terrestrial open habitat. While undertaking long—and long-distance—bouts of bipedal locomotion, distributed cognitive management frees up other embodied cognitive systems for visual, auditory, and olfactory perception, semiotic construction, planning, sustained goal-oriented selective attention, and active communication (cf. Langdon, 2005).

### The embodied bipedal terrestrial niche: reduced social alertness during foraging

Hominin locomotion adaptations emerged from ca. 7–5 million years ago (mya) onward. Their evolution initially co-occurred with the phylogenetic divergence of our lineage from that of chimpanzees and bonobos (Won, 2004; Lovejoy, 2009; Lovejoy et al., 2009; Webster, 2009; Yamamichi et al., 2011). In early hominin populations directional selection modified the ancestral ape pattern of quadrupedal walking and vertical climbing (Sockol et al., 2007). Here, the evolutionary process would have favored locomotor phenotypes that were not only generally fit to the mosaic forest-grassland habitat features of East and northern Central Africa, but also minimized energy expenditure in that habitat (Wheeler, 1991a,b; Leonard and Robertson, 1997). The emergence of the genus *Australopithecus*, ca. 4 mya in East Africa, appears to have coincided with the evolution of “obligate bipedalism,” in which the anatomy supporting efficient stride is so specialized that it substantially limits habitual arboreal climbing (Jungers, 1982; Latimer et al., 1987; Latimer and Lovejoy, 1990; Ohman et al., 1997; Haile-Selassie et al., 2010). Obligate bipedalism—involving an arched foot, non-opposable big toe, and a strongly disto-medially angled femur—is first documented among fossil traces of *Au. anamensis* (ca. 4.2–3.9 mya) and *Au. afarensis* (ca. 3.7–3.0 mya) in East Africa. In modern humans obligate bipedalism adapts our bodies highly efficiently to moving around, slowly but surely, in a terrestrial diurnal habitat, during continuous trips that may last hours and cover as much distance as the diameter of some wild chimpanzees' and gorillas' lifetime territories (ca. 30–50 km). Although australopithecines exhibited a range of vertical climbing and pedal locomotor grasping anatomy (DeSilva et al., 2013), skeletal support for bipedal locomotion in *Au. afarensis* (a fossil species most famously represented by the partial skeleton “Lucy,” specimen AL-288-1) had already evolved as an integrated adaptive system between ca. 4–3 mya, well fit to terrestrial activity (Haile-Selassie et al., 2010).

As such, early obligate bipedalism was a *phenotype* shaped by natural selection in a long-term process of niche-population co-evolution. Yet, as a *niche component*—that is, as an embodied interface with a terrestrial, relatively open habitat that housed a very diverse, heterotrophic food resource spectrum—obligate bipedal locomotion entailed new selective pressures. First, obligate bipedalism exposed early hominins to a range of large felid predators (Hart and Sussman, 2011). As a phenotype in a foodweb heavy with large carnivores, bipedal sprinting is no match for well-adapted quadrupedal running over short distances (Leonard and Robertson, 1997). It may be inferred—based on theoretical models and comparative data (Sussman et al., 2005; Hart and Sussman, 2011)—that during this critical period of niche-population co-evolution, natural selection would have favored pro-social behaviors for aggregation. Being part of a group not only enriches the individual's perceptual information about predatory threats, via indexical predator alert calls (Seyfarth et al., 1980; Zuberbühler, 2001; Stephan and Zuberbühler, 2008). It also simply reduces the likelihood that a given individual will be the one ambushed and captured by a lion or sabretooth cat hiding in the tall grass (cf. Hamilton, 1971). The bipedal embodied niche initially involved, at the very least, frequent bouts of reduced social attentiveness, coupled with increased alertness for indications of diverse predators—and of prey. Yet, when terrestrial foraging was successful, the immediate extrasomatic environment would have changed dramatically. If the discovered food resource was rich enough—whether it consisted of larger game or of carbohydrate-dense or fatty plant tissues—cooperatively transporting and defending that food resource would have tended to increase the group-members' average inclusive fitness. Here, distributed cognitive management of locomotion would have been especially important. Individuals would have had to carry bulky or heavy food packages, while suppressing attention to strong emotional-desire responses to hunger sensations, with heightened, rapidly shifting attention on immediate group members, predator risks, and near-term future possibility for satiating hunger in a safer aggregation locality.

### The embodied bipedal terrestrial niche: cooperative offspring care and intertwined narrativized social identities

Obligate bipedalism—as an embodied niche interface system—thus conspicuously supported pro-social adaptations that went beyond simple gregariousness. In fact, there were profound social implications for hominin bipedal locomotion in a terrestrial, heterotrophic niche. Other things being equal, bipedal anatomy is not only relatively maladaptive for escaping large quadrupedal carnivores. For the adult female, it may be considered an evolutionary compromise—in comparative primate evolutionary ecological perspective—for giving birth (Tague and Lovejoy, 1986; DeSilva, 2011; Kurki, 2011, 2013; Wells et al., 2012) and carrying relatively helpless infants (Wall-Scheffler, 2012), while also supporting long bouts of bipedal walking. Pelvic morphology well suited for lowering the body's center of gravity and reducing mechanical effort (and short-term metabolic balance and long-term stress on muscles, bones, and associated connective tissues) during upright walking exhibits a limited pelvic aperture (Lovejoy, 1988; Rosenberg and Trevathan, 1995). Natural selection has shaped this functional anatomical compromise in the adult female hominin body, achieving efficient bipedal stride at the expense of more frequent, riskier obstetric complications giving birth to large-brained neonates. Given the evolutionary success of this compromise—measured in terms of extant human geographic range and biomass—it is clear that the typical population-level survival and reproductive success benefits of bipedal locomotion have more than made up for any morbidity and mortality risks associated with parturition through a bipedal pelvis.

The realized reproductive success associated in integral part with bipedalism is all the more remarkable, because the posture requires adults to use their arms to carry infants and young juveniles, raising the adult's center of gravity during locomotion. This imposes a relatively greater metabolic and stress cost in caring for very young offspring who are still developing sufficient fine and gross motor strength and coordination. Without a compensating phenotype, the “carrying cost” would increase risk of predation for mother and infant, alike. It would also reduce her foraging efficiency, slowing down food search rates, while burning more calories to search for food. The resulting metabolic deficit would also increase mortality risks for a lactating mother and her offspring. Virtually the only theoretically plausible behavioral phenotypic compensation that would have co-evolved with the bipedal embodied niche is alloparenting: cooperative offspring care.

**Figure 4:**
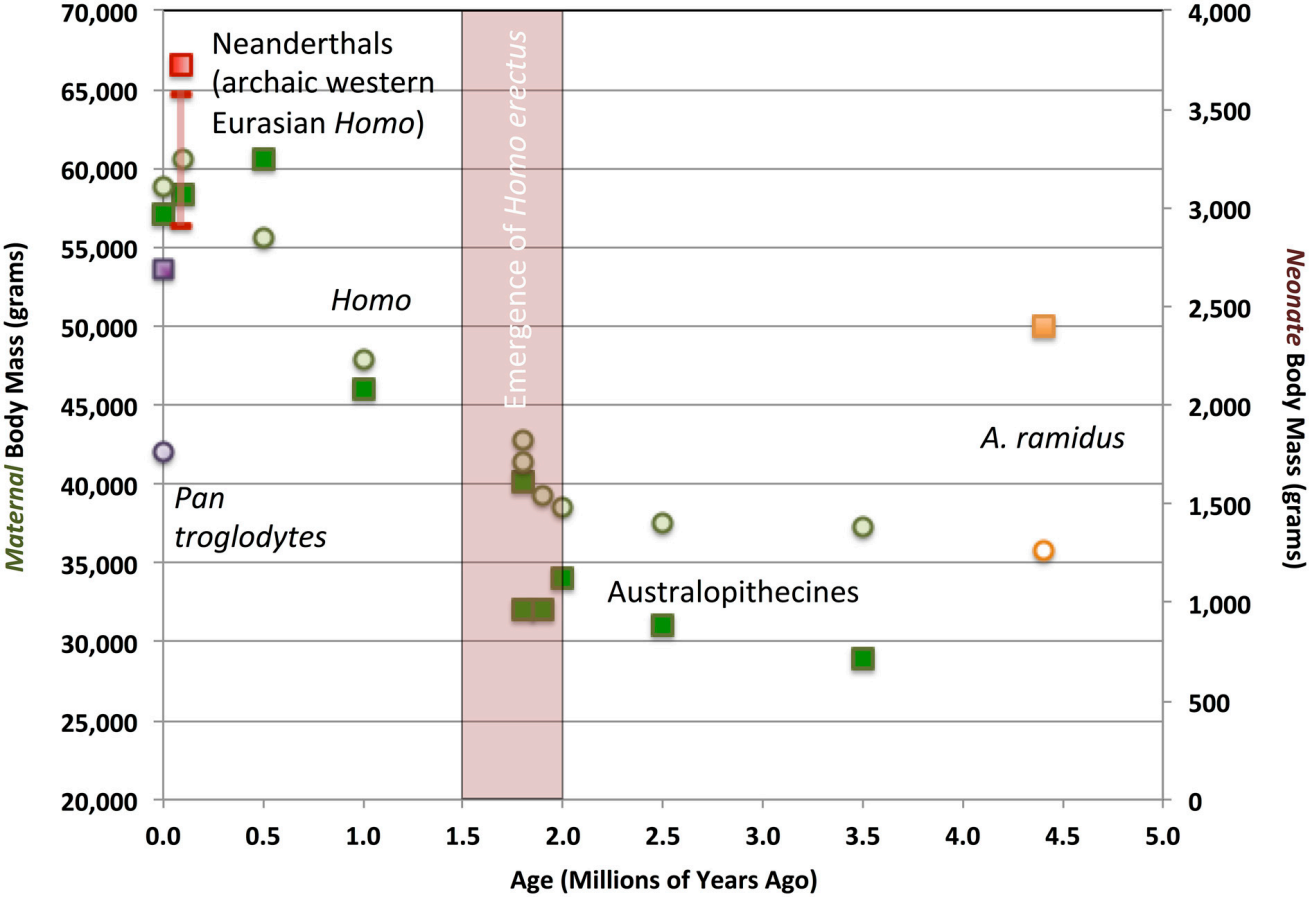
**Evolutionary change in maternal and neonate body mass in the hominin lineage, compared with extant chimpanzees**. Maternal body mass averages measured from fossil and modern samples shown as squares. Neonate body mass averages measured from fossil and modern samples shown as circles. *Ardipithecus ramidus* shown in orange. *Australopithecus* and *Homo* samples shown in green, except for Neanderthals, shown in red. The Neanderthal female and neonate fossil samples raise the possibility that this late Pleistocene (ca. 200-40 thousand year old) western Eurasian population may have evolved a higher body-mass:brain ratio, compared to contemporaneous regional human populations (VanSickle, 2009; DeSilva, 2011). The Neanderthal neonate body mass estimate is thus shown as a conservatively estimated range. Modern chimpanzees mothers and neonates are shown in purple. Modern and fossil maternal body mass measurements and estimates are from VanSickle (2009) and DeSilva (2011), and neonate body mass estimates are calculated after the methods in DeSilva and Lesnik (2008).

**Figure 5:**
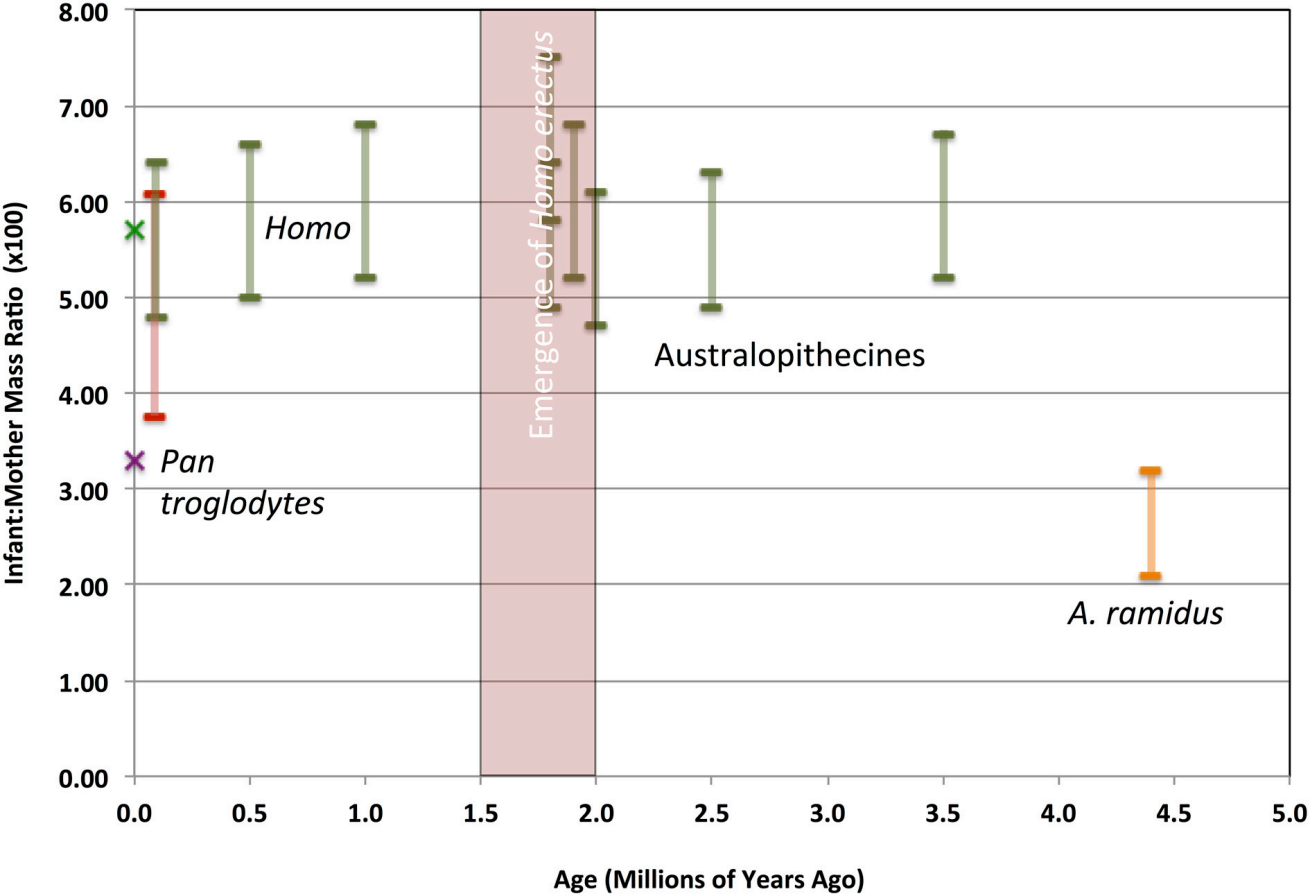
**Evolutionary change in neonate:maternal body mass ratios in the hominin lineage, compared with extant chimpanzees**. Samples are color-coded as in Figure 4. Fossil hominin samples are shown as estimate ranges. Extant human and chimpanzee sample means shown as ×s.

Indirect, circumstantial evidence for alloparenting primarily consists of data on the metabolic costs and ecological risks otherwise imposed by carrying infants and young juveniles during bipedal locomotion. Further evidence comes from estimates of adult maternal and neonate body mass^1^. Figure 4 depicts variation in maternal and neonate biomass in hominin samples distributed across the last 4.4 million years of our lineage's evolution, mainly based on data from DeSilva (2011). The samples begin with *Ardipithecus ramidus*, fossil specimens of which document a hominin population lineage that had retained the ancestral anthropoid opposable big toe, along with other anatomical indicators of “non-obligate” bipedal locomotion and vertical climbing adaptations in a forested East African habitat (Lovejoy, 2009). In comparison, the later hominin samples from 4.0 to 2.0 mya include extinct or ancestral australopithecine species, whose fossil remains document early obligate bipedal adaptations. As DeSilva (2011) has emphasized, a substantial evolutionary reduction in adult female body mass occurred from *Ardipithecus* to *Australopithecus*. That such a shift occurred early in hominin evolution was not apparent prior to publication of the description of *A. ramidus* post-cranial anatomy (Lovejoy, 2009; Lovejoy et al., 2009). *A. ramidus* females (represented by the remarkably intact adult female individual “Ardi,” whose estimated body mass is represented in Figure 4 by the orange square) had an adult size comparable to the typical level in living chimpanzees (ca. 50 kg, also shown in Figure 4, represented by a purple square). Later, australopithecines and early members of the genus *Homo* inherited modified, reduced female body mass, with adults weighing only 30–40 kg. Yet, small adult female body size was linked to a substantial jump in encephalization, because head-size was as large or larger than that seen in apes and ardipithecines. Australopithecine and earliest *Homo* adult endocranial volumes (both sexes included) spanned ca. 400–800 cc. The lower end of this range overlaps with the upper end of the chimpanzee distribution. Thus, the slight australopithecine maternal body mass values would have been associated with relatively large—and large-brained—neonates. Exhibiting obligate bipedal anatomy, the small australopithecine/early *Homo* maternal body would have contributed to high neonate:maternal body mass ratios (Figure 5). This constitutes additional, albeit indirect, evidence that alloparenting co-evolved with bipedal locomotion. The actual maternal costs of carrying an infant while walking upright were greater than paleoanthropologists have previously thought. Evolutionary theory predicts that, other things being equal, early hominin mothers with either more evolutionarily ancestral (i.e., ape-like) or more derived (i.e., like large-bodied *Homo*) body mass and locomotor anatomical traits would have had a reproductive-success advantage in mosaic East African forest/grassland habitats. Yet, the smaller, more vulnerable, nutritionally precarious australopithecine maternal bodies should have faced stiff evolutionary competition. The australopithecine/early *Homo* pattern—small maternal bodies and high neonate:maternal body mass ratios—contradicts such standard evolutionary expectations.

This development may be explained by australopithecine co-evolution with alloparenting behaviors, associated with a female life-history strategy that gave up adult somatic mass, in order to maintain caloric and nutrient resource transfers to offspring during gestation and lactation. Small body size would have constituted a costly, honest signal that a mother actually needed assistance in carrying or provisioning offspring in order to maintain energy balance for herself and for her infant.

Arguably, then, the question is not whether alloparenting co-evolved with obligate bipedal locomotion and terrestrial foraging/costly infant-carrying in australopithecines. It is rather: What was the social structure of alloparenting, particularly in relation to courtship, mating, and possible cooperative foraging or food-sharing behaviors (Hrdy, 2009)?

**Figure 6:**
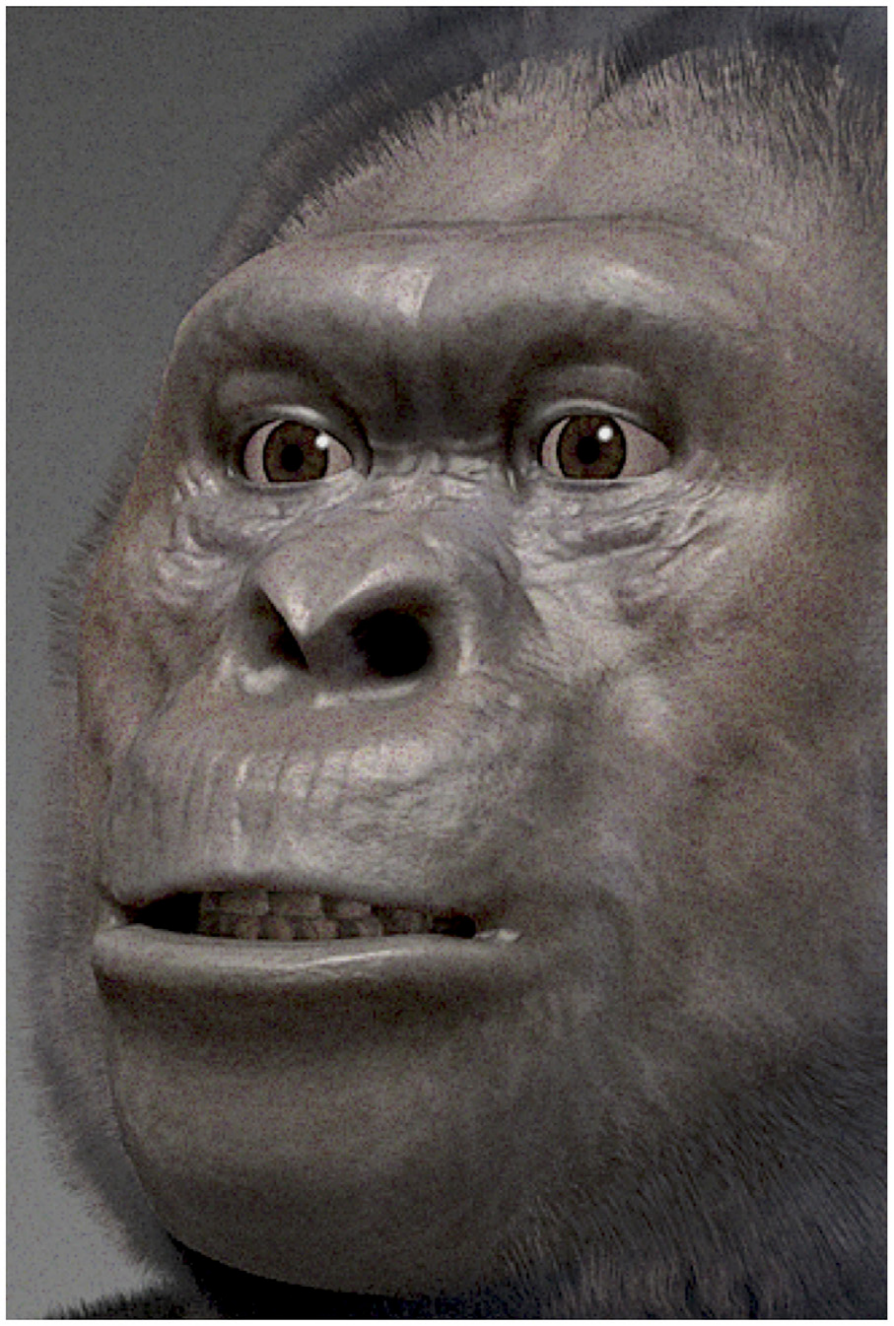
**Forensic reconstruction of a male *Australopithecus afarensis* adult**. The male and female *Au. afarensis* permanent dentition exhibits smaller canines than seen in *Ardipithecus*, suggesting evolution of honest signaling of reduced fighting ability. Photograph accessed from http://upload.wikimedia.org/wikipedia/commons/2/22/Australopithecus_afarensis.png.

Here, I present a plausible theoretical claim for the following structure in early australopithecine social systems. Alloparenting would have fundamentally involved unrelated females—having transferred at maturity from their natal groups, as observed in living gorillas, chimpanzees, and bonobos—engaging in reciprocally altruistic assistance during parturition and early childrearing. Continuous honest signaling of maternal and infant need—in the embodied form of mother's small size—would have also favored reduced male aggression and male provisioning of offspring. This is because adult males would likely have faced a time and energy trade-off between provisioning mates and maintaining and defending a female harem. From this perspective alone, it may be argued that bipedal locomotion in a terrestrial, heterotrophic niche would have favored reduced male aggression, evidence for which may be seen in the substantial evolutionary canine-size reduction seen in males and females alike, from ca. 6–4 mya (*Ardipithecus*) to ca. 4–1 mya (*Australopithecus* and early *Homo*) (Figure 6) (Haile-Selassie and WoldeGabriel, 2009). In fact, the most effective strategy for repeatedly obtaining additional calories, in the form of divisible “public-goods-like” food packages (cf. Hawkes et al., 1998; O'Connell et al., 1999, 2002; Hawkes, 2003) that could be shared with mates and (statistically likely) offspring—or at least brothers' or half-brothers' offspring—would be for males to cooperate, at least occasionally, in foraging, food transport, and food sharing.

Thus, three interrelated social behavioral patterns would have defined the embodied aspects of the obligate bipedal niche: adult female reciprocal cooperative alloparenting (and—possibly—midwifery); male cooperative foraging and food transport; and male provisioning of mates (Table 1). This behavioral nexus would have constituted a dynamic interface between the body and a socially intense, yet strongly ecologically structured extrasomatic environment.

**Table 1:**
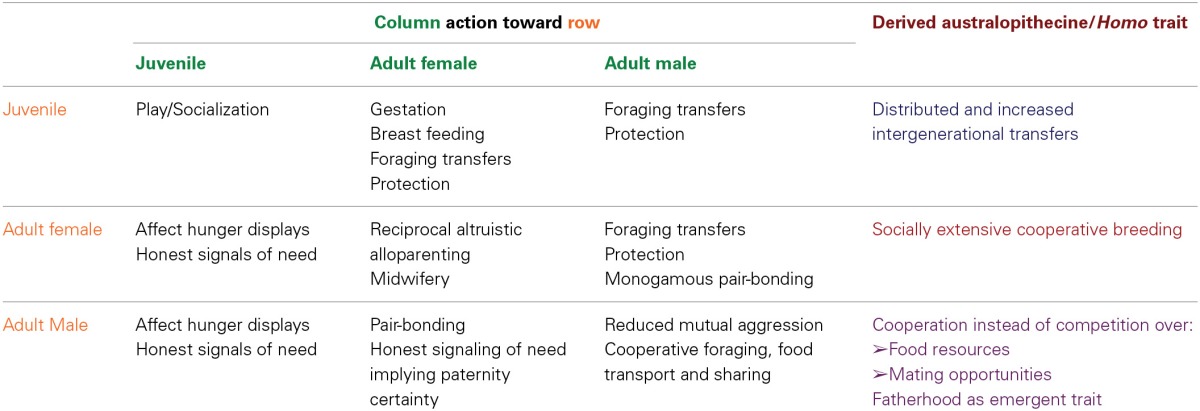
**Matrix of predicted social interactions among adult males, adult females, and juveniles in *Australopithecus afarensis*, based on the embodied niche construction hypothesis**.

The ENC hypothesis supports a key prediction: that long-term monogamous pair bonding would have also co-evolved with the bipedal niche, albeit hardly strictly driven by active male foraging, resource transport, and provisioning of females (cf. Lovejoy, 1981, 2009; see Table 1). To be sure, from the adult female's perspective, the socially intense bipedal interface would have partially structured—and been structured by—females' continuous honest signaling to males of precarious energy balance and risk of predation. Critically, this would have occurred across three key foci of adult female social attention:

- courtship
- soliciting food transfers or physical protection during gestation and lactation
- continued food transfers, protection, and learning support for weaned subadult offspring

Yet, the ENC hypothesis predicts that adult males and females would likely have developed complex pair-bonding relationships through recurrent bouts of food transfer solicitation, food transfers, courtship and sex, separating and reuniting, and mutual monitoring during group travel or nighttime aggregation. This social interaction and monitoring would have supported higher cognitive construction of mutually indexical, highly emotional iconic narratives (see Figure 3) of solicitation and resolution, jealousy and relief, and (often substantially prolonged) anticipation and satiation. Semiotic construction of simple mental iconic narratives would have been recurrently evoked through perceived embodied experiences. These iconic representations would have been constructed in part through indexically evoked similarities and contrasts with two other “australopithecine genres” of iconic narratives: those dealing with reciprocal social assistance—including midwifery—between adult females and those involving cooperative foraging and food transport among adult males. In the context of female-male pair-bonds, these evoked narratives would have indexically focused the couple's joint attention on circumstances at hand that would afford actions—ranging from traveling together, to mutually caring for offspring, to grooming, and to sexual intimacy—that would reinforce the pair bond.

### The embodied bipedal terrestrial niche: infant helplessness and dialectical cognitive construction of self and other

Obligate bipedal anatomy seems especially maladaptive for the australopithecine infant (other things being equal, of course). To be sure, cooperative social networks would have supported protecting, transporting, and provisioning infants. And in fact, this would have more than compensated for the fact that the especially vulnerable human infant is born with undeveloped, only potentially supportive anatomy for bipedal locomotion. The evolutionary loss of grasping toes in *Australopithecus afarensis* (ca. 4–3 mya) and later hominins (ca. 3 mya and onward), coupled with the mother's habitual upright stance, would have rendered the infant especially dependent on the parent. The infant could do little—in terms of motor behaviors—to minimize risk of separation. Thus, accidental separation could result in accidental abandonment.

Moreover, the infant's embodied sensory-motor and visual interface with the upright bipedal mother entailed new challenges for learning in early childhood. Bipedal balance and locomotion likely imposed a steeper embodied cognitive learning curve than would the ancestral quadrupedal walking/grasping/climbing locomotor pattern. It is simply harder to learn to balance on two legs while standing upright than it is to balance on four limbs.

The mother's bipedal posture and gait also combined with her infant's very limited ability to grasp. This would have prevented the infant from habitually orienting its body in parallel to the mother, while lying on top of the mother's back. In apes and monkeys, the highly effective and sensorily rich activity of grasping onto mother's back synergistically allows the infant to feel the oscillatory rhythm of walking on all fours, while also resembling quadrupedal grasping during arboreal climbing. In australopithecines and early *Homo*, in contrast, the earliest experience of being carried during travel and foraging would have been deictically lateralized. Consequently, the obligate bipedal infant would have had a relatively distorted embodied experience of the rhythms of habitual locomotion.

#### The embodied narrative of exerting agency

Whereas riding on mother's back incrementally prepares the non-human primate infant to separate from the mother and actively explore, the human infant has a very different embodied learning experience. During the first months of life, the human infant registers and learns about the world through visual, auditory, gustatory, and olfactory inputs. Yet, gross motor balance and fine haptic inputs—which provide short-term, continuous feedback during active embodied learning in non-human primate infants—would have been severely limited for australopithecine and early *Homo* neonates. Thus, the initial period of hominin learning is frequently shaped by a passive embodied interface with the extrasomatic environment. Gross motor strength and balance gradually develop—through learning to lift up the head, roll, sit, crawl, stand, and walk. For the human infant, this embodied learning occurs in extrasomatic environments defined by a protective, usually taken-for-granted adult social network. Still, as I have underscored above, the infant cannot efficiently use embodied memory of earlier experience of being carried by an adult, as she begins independent gross motor learning. This early learning experience, then, can canalize construction of a heroic iconic narrative of exerting agency and achieving greater control over the environment.

#### The embodied narrative of forming judgments

Moreover, this iconic narrative would be indexically linked to another, contrasting early-life iconic narrative, in which the infant observes the extrasomatic environment over extended bouts, perhaps as long as an hour, at a distance. The infant is alert but passively secure, able to focus on objects and events around her. This iconic narrative would be one in which passive monitoring results in changing affect. The infant can look, hear, smell, taste, and *judge*.

#### Overview of iconic narratives in the obligate bipedal niche: ideology and praxis

The two main learned iconic narratives of the first months of life are then speculated to be those of exerting agency, on the one hand, and of judging the situation, on the other. I argue that the obligate bipedal niche itself would have encouraged the embodied learning of reflexive alertness in the first months of life, even prior to natural selection for derived higher cognitive phenotypic functions.

When the hominin juvenile has attained sufficient motor strength and balance to begin interacting with a wider environment over longer intervals, she experiences a major contrast between the more passive infant interface and the new, highly exciting but difficult juvenile one. The young juvenile's embodied capital now affords it the agency to seek out and integrate haptic and motor information with other sensory inputs. Here, new iconic narratives become indexically linked to the early-life narrative of agency, in turn, contrasting with the early-life narrative of affective judgment. This may be seen as the ontogeny of an individual dialectic between ideology—the semiotically constituted, narrativized representations of how one hopes or would like the world to be—and praxis—that is, what one does, or does not do, to actualize that ideology.

### Overview of embodied niche construction through the bipedal interface

The bipedal niche would have minimally supported embodied pro-social bonding between juveniles and their maternal relatives, between adult females, and between adult males (see Table 1). The ENC hypothesis deepens existing ecological explanations of how obligate bipedalism evolved. This phenotypic system is traditionally seen strictly as adaptive to a primarily terrestrial heterotrophic niche in East and north Central African, initially among australopithecine populations, ca. 4–3 mya. The dynamic co-evolutionary model of reciprocal causation in niche and population change—based on niche construction theory—illuminates the possibility that obligate bipedalism as an *embodied niche* ontogenetically structured a semiotic interface with the extrasomatic environment. This interface mediated sustained embodied attention on prevailing social and material situations, through constructed, indexically interrelated iconic narratives.

The ENC hypothesis predicts that sustained introspective attention and more hierarchically complex semiotic systems co-evolved with bipedalism. Both semiotically structured cognition and bipedal locomotion were phenotypic adaptations and embodied niche interfaces. The complex niche-population co-evolutionary dynamic resulted in overall population fit to a much more open, yet dangerous terrain, but supported by a rich and diverse range of heterotrophic food resources. Thus, the ENC hypothesis further predicts selection for aggregating in large groups to avoid predation—especially from evening until morning. It also predicts alloparenting by older juvenile and adult female maternal allies, adult female mutual assistance during childbirth, and group fissioning during diurnal, omnivorous, and nonetheless gregarious—if not outright altruistic or synchronized cooperative—foraging, followed by occasional food transport and group fusion. The semiotic component of the embodied bipedal niche is also predicted to have favored the evolution of more complex symbolic thought, which would have sustained the individual's attention on multi-step longer-term (at least hour-scale) planning, delaying gratification, and making social judgments in guiding solicitation of help, offers of assistance, and mobilization of cooperative activities.

## Discussion

The hominin (human) and panin (chimpanzee and bonobo) lineages mutually evolved a reproductive barrier during the late Miocene period, ca. 7–5 mya, in Subsaharan Africa. The foodweb and habitat features that distinguished hominin niche construction from that of the panins included a spatially more extensive terrestrial setting and a broader heterotrophic prey spectrum. The case study on early australopithecine obligate bipedalism, ca. 4–3 mya, supports the argument that hominin niche construction further differed from that of the panins, increasingly involving semiotically structured cognition in the embodied bipedal interface. This interface facilitated adaptive behavior in an intensely social and complex material habitat.

According to the most recent paleoanthropological research, the final Pliocene and early Pleistocene periods—roughly 2.5–0.8 mya—encompassed even more dynamic co-evolutionary change in the embodied niche. The oldest known stone tools were used early in this time frame, with the emergence of the genus *Homo* following soon afterward (Kimbel et al., 1997; Semaw et al., 1997; Semaw, 2000; Domínguez-Rodrigo et al., 2005; Stout et al., 2005; Goldman-Neuman and Hovers, 2009; Kimbel, 2009). We can infer further change in the bipedal interface, which exhibits interrelated modifications in balance, static upper-limb loading, visual, and auditory components. Changes in the anatomy of the hand and thumb supported precision grip in early *Homo*. This adaptive modification would have been integral to making and using small stone tools with the thumb and other manual digits (Marzke, 1997; Marzke and Marzke, 2000). The resulting joint visual and fine manual interface would have structured bouts of goal-oriented, highly visually focused interaction with the material environment (Stout et al., 2008; Stout, 2011), suppressing embodied attention toward social or wider environmental information. Such sustained goal-oriented embodied attention would have contrasted with embodied experiences during which partially decentralized cognition supported locomotion or rhythmic, repetitive tool-making or use, while also maintaining attention on surrounding social events, facilitating social judgment. ENC thus favored temporally alternating interfaces involving social alertness suppression and very context-sensitive social hyper-alertness.

Hominin ENC was especially shaped by those behavioral traits—including bipedal locomotion, tool-making, resource transport, food processing and social food consumption (involving non-agonistic interactions during feeding, as well as active sharing)—whose long-term evolution already had come to define a distinctive adaptation-niche coevolutionary trajectory. These key behavioral phenotypes, whose evolution was well underway by the Middle Pleistocene period (ca. 780-130 thousand years ago [kya]), were also components of the hominin niche. The Middle Pleistocene timeframe is important as we begin to consider how the ENC hypothesis might inform speculation, hypothesis formation, and research methods concerning the evolution of human language. Toward the end of the Middle Pleistocene, extant absolute brain volumes emerged in Subsaharan African anatomically modern humans (AMH) and western Eurasian Neandertals (ca. 200–130 mya) (Lee and Wolpoff, 2003). In addition, extant body proportions—including encephalization quotients—appeared in early AMH populations, after ca. 200 kya (Ruff et al., 1997). The embodied niche complex that co-evolved with the australopithecines and early genus *Homo*, then, was not dependent on a modern-sized brain or brain-body proportions. Evidence on skeletal growth rates and aging in fossil remains from the entirety of the Pleistocene era (ca. 1.80–0.01 mya) further suggests that initial adaptation to the early *Homo* embodied niche complex was not dependent on modern patterns of slow, delayed maturation and significant post-reproductive survival (Caspari and Lee, 2004, 2006; Smith et al., 2007a,b).

Here, the ENC hypothesis can elegantly explain the further coevolution of embodied niche with a hypothetical proto-language capacity (cf. Tomasello, 2008). The embodied dimension of the emergent hominin niche would have favored long-term selection for formally simple—and likely declarative but implicitly deictic—gestures or utterances that constituted symbolic representations. Such utterances would have been experienced as embodied sensory-motor concepts, whether they were initially verbal or brachial-manual gestures. Already defined by a complex web of visual, auditory, and motor-simulation associations that comprised a socially learned proto-language, the earliest verbal symbols would have further pointed to states of affairs in the environment, indexically tying together somatic and extrasomatic aspects of the prevailing milieu (Gallese and Lakoff, 2005; Tomasello, 2008). In turn, this would have synergistically reinforced learning complex foraging behaviors and social competence in networks sustained by strong reciprocity/reputation monitoring (Bowles and Gintis, 2004; Nowak and Sigmund, 2005). Linguistic communication would have shared a fundamental structural and functional similarity with omnivorous foraging, tool-making, and social cooperation (Stout et al., 2008). These embodied cognition/environment interfaces basically involve sustained attention on representation-dependent, goal-directed activity sequences, which may unfold over minutes or hours—with further potential for resuming attention on representation-dependent activities after intermittent breaks. Such embodied cognitive attention is hypothesized to reduce immediate sensory alertness levels, because the higher-level cognitive processes go beyond sensoro-motor cognitive simulation, involving more complex abstract narrative construction.

In light of the paleoanthropological evidence, the ENC hypothesis entails two mutually exclusive—but nonetheless plausible—evolutionary trajectories:

- Linguistic utterance and comprehension co-evolved gradually with the genus *Homo's* embodied niche—following the establishment of the bipedal interface and the subsequent emergence of the manual precision/tool-making/tool-using interface—beginning as early as 3.0–2.0 mya (Schepartz, 1993; Deacon, 1998; Lieberman, 2013); or
- Spoken language rapidly evolved more recently (Chomsky, 1986), preceded by a long period of evolution in mental narrative construction and selective introspective attention—only later co-evolving with extant biological life history patterns of growth, maturation, aging and mortality (cf. Caspari and Lee, 2004, 2006; Smith et al., 2007a; Smith, 2013).

In either case, the evolutionarily derived hominin capacity for *narrativizing* simple iconic or symbolic semiotic representations would have evolved through by natural selection during the Pleistocene era. As argued throughout this hypothesis and theory article, it is predicted that the capacity to construct—and even recursively share representations of—embodied, iconically and symbolically represented narratives would have emerged via the dynamic bipedal and social monitoring/judgment interfaces that centrally define our terrestrial, extractive, and socially intensive extrasomatic environment.

### Narrative representation as embodied temporality

Theoretical approaches to human and non-human animal learning and behavior still strongly emphasize synchronic embodied representation or near-instantaneous cognitive feedback. Philosophers of cognition, experimental psychologists, and brain imaging experts have convincingly explained that embodied simulation connects affective and proprioceptive states or motor memories to extrasomatic stimuli (Barsalou, 1999, 2009; Damasio et al., 2004; Gallese and Lakoff, 2005; Heyes, 2010a,b; Dove, 2011; Man et al., 2012). Moreover, recurrent memory construction surely often shapes perceived extrasomatic phenomena or body-environment relationships, which specifically have a synchronic or short-term antecedent-consequent structure. Such embodied perceptual concepts may range from learning relevant, albeit complex figure-ground contrasts to more comprehensive, amodal learning of predator threat concepts or subtle indices of prey availability. Mapped onto Deacon's (1998) semiotic framework, such embodied concepts develop through bodily interfaces with the surrounding environment—specifically as *stable indexical relationship systems*. Cognized objects or bodily states immediately point to—or are pointed to by—other objects or somatic states.

If I am right that the embodied, private iconic narrative constitutes a key adaptive phenotype/embodied niche component in hominin evolution, then it would be especially important to consider the *narrativized temporality* of such indexical relationship systems. Quite different perspectives in recent human cognition scholarship have converged on highlighting embodied narratives and cognitive sensitivity to temporal duration and degrees of pastness (Hutto, 2008; Menary, 2008; Gallese, 2011; Panksepp and Biven, 2012). So far, I have emphasized two embodied iconic, non-linguistic narrative genres as important for the ENC hypothesis (for details, see section The Embodied Bipedal Terrestrial Niche: Infant Helplessness and Dialectical Cognitive Construction of Self and Other). The first is the exertion of agency, which is a narrative of self having—or failing to have—an effect on someone or something. The second is the achievement of judgment, in which self achieves—or fails to achieve—an unambiguous affective disposition concerning someone or something else that she has been regarding in the extrasomatic environment. Such simple metacognitive narratives would have a scale-free, relative temporality: before, the situation was ambiguous or uncertain, but now, self has achieved a clear outcome. The relative chronological structure of these genres would reflect the most basic, temporally marked indexical relationship. On the one hand, a representation of the earlier, ambiguous past can point toward memory of reaching a clear outcome in the relatively more recent past. On the other hand, a prior representation of the remembered or imagined, more recent clear outcome could evoke the entire (possibly tragic, possibly triumphant) narrative arc, from earlier ambiguity to subsequent clarity. The very general, scale-free structure of the agency and judgment narratives has a series of remarkable implications:

➢ Iconic narrative representations may initially evoke the general, familiar feeling of attention on self's body-environment interaction, as it unfolds over short timescales, from fractions of a second to hours. Yet, they may recursively evoke a chain of chronologically ordered, nested, or longer continuous stories of agency or judgment. As such, the iconic narrative comprises the formal semiotic building block for representing diverse, complex pasts.
➢ The generic, elemental narrative form efficiently contextualizes and emphasizes diachronic changes in self's affective emotional states. As such, self's selective introspective or mindful attention on narrativized memories can autocondition complex learning. This may be especially important for developing competence at intricate, temporally extended, choppy, and contingent behavior sequences in the hominin terrestrial, extractive, heterotrophic, and intensely social niche.
➢ Simple iconic social judgment narratives have a particularly strong general conditioning effect on building complex stable indexical relationship systems. When self learns embodied understanding of a social other, her embodied cognitive construction process sets up a hierarchically constituted triadic association—one that is very familiar to structural anthropology, in which two terms are equated or opposed, and in turn, that dyadic relationship is metonymically indexed to a third, generally evocative term (Lévi-Strauss, 1963; Leach, 1976). Here, the socially interacting or regarding agent perceives that the other is either similar or dissimilar to herself, in some markedly evocative respect. Her embodied experience provides rich synchronic information, involving her own perceptions of the grounding environment in which the other is defined; her prevailing proprioceptive or interoceptive states; and memory content evoked by the situation at hand. This embodied information is material for reinforcing or metonymically tagging the experience that other is—or is not—like self. As an embodied concept that integrates perception of other with evoked, sensory-motor/emotional simulated experience, the understood relationship between self and other has its own rich potential to evoke—that is, to approach synchronic juxtaposition with—learned concepts that have similar experiential tags or indices.
➢ The auto-indexical connection between the narratives and the emotional changes embedded in them further recursively rewards introspection and the mental consideration of plausible and implausible associations, alike, so that narrativized embodied representations mediate changes in self's affective states over time.

In general, through recursive iconic narrative construction, embodied cognition can “pancake” or elide diachronic features of the narrative's content, yielding new synchronically represented associations among action sequences, changes in the extrasomatic environment, and changes in bodily affective states (Table 2).

**Table 2:**
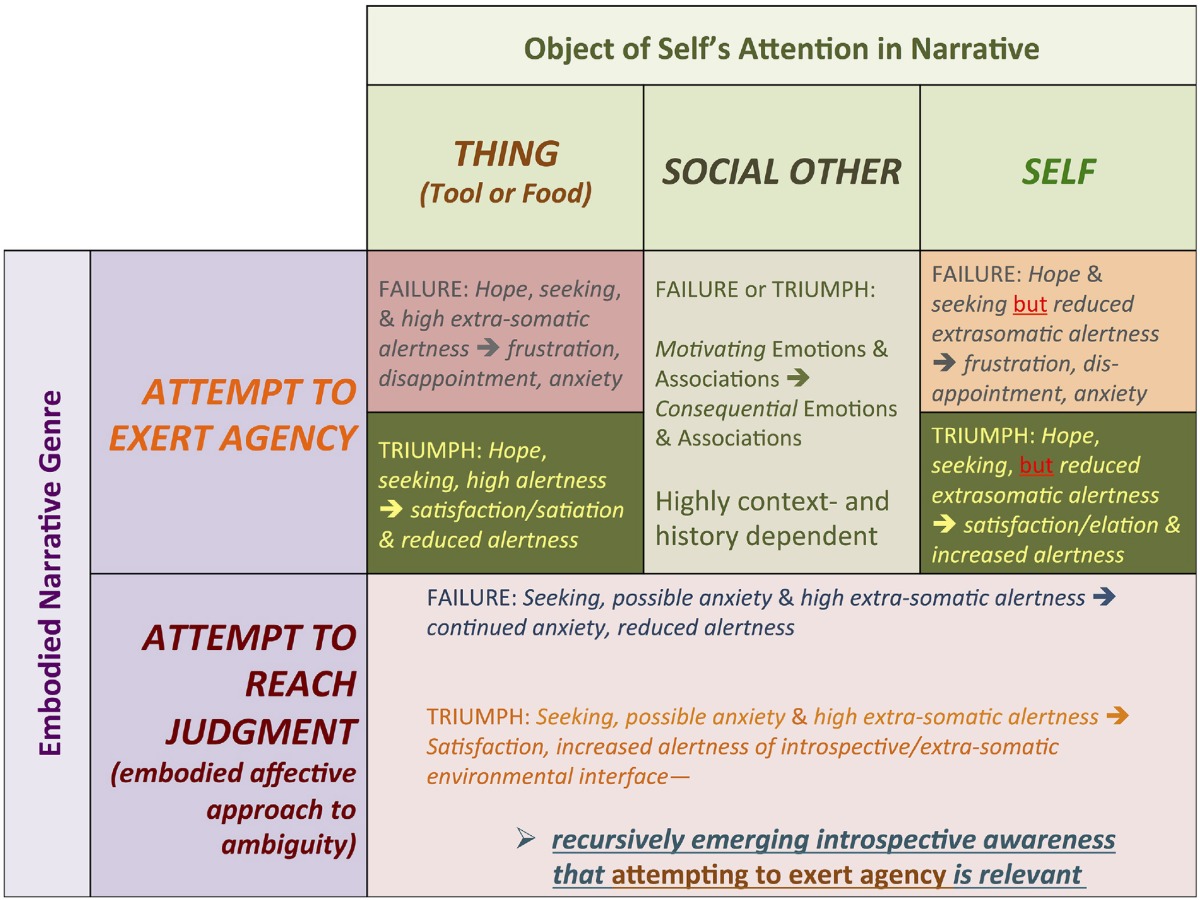
**Matrix of hypothetical emotional and affective trajectories of the two main proposed genres of embodied iconic narratives emerging in human evolution**.

### Iconic narratives as evolutionary precursors to socio-linguistic constructions

The theoretically plausible—albeit preliminary and speculative—argument for the ENC hypothesis, presented above, also implies the following. As joint adaptive phenotype and embodied niche component, *recursive iconic narrative construction* was evolutionarily ancestral to natural open language systems, with their fundamental feature of “double articulation.” Logically consistent patterns governing the orderly juxtaposition (that is, the articulation) of message features require that similar, substitutable elements—on the dual, nested levels of phonological regularities and syntactical structures—are available (Jakobson and Halle, 1956; Hockett, 1961; Lévi-Strauss, 1963; Saussure, 2011). Double articulation is an apparent formal and functional necessity for natural languages. More generally, though, interaction between message-feature *contiguity* and *similarity* makes double articulation possible (Jakobson and Halle, 1956; Lévi-Strauss, 1962a). The recursive interplay between contiguity and similarity is, in turn, sufficient to generate the openness of natural language, in which a finite set of signs may be combined, repeated, and substituted to generate potentially infinite expressions or representations (Chomsky, 1986; Hauser et al., 2002). Thus, contiguity-similarity interaction can structure and be structured by hierarchically nested message elements, in which one or more element-levels incorporate coherent, independent messages. In other words, double articulation at the phoneme and lexico-grammatical levels may be understood as an instance of our more general capacity for embodied cognitive recursion—a capacity that emerged gradually in hominin evolution, beginning well prior to language evolution itself.

### From theory to practice: anthropological perspectives on testing the ENC hypothesis

The ENC hypothesis has particular potential to connect general anthropological (including ethnographic), paleoanthropological, cognitive science, and comparative experimental psychological approaches to human cognition and embodied experience. My aim in this article has been to outline the ENC hypothesis in some detail and attempt to establish its relevance for explaining the evolution of human cognition over very long timeframes. This simply reflects my paleoanthropological research specialization. However, my hope is that the ENC hypothesis can guide collaboration among anthropologists, cognitive scientists, and experimental psychologists, redefining and expanding theories, evolutionary perspectives, and observational and experimental designs. We should be able to evaluate whether the ENC hypothesis provides a more reliable, comprehensive explanation of how abstract or “dis-embodied” concepts (Dove, 2011)—as dynamic features of our semiotically structured worlds—might arise from, yet remain grounded in indexical associations with embodied sensory-motor representations and iconic memories. Perhaps the most concrete, testable prediction of the ENC hypothesis is that structured measurable changes in affect, emotional state, and sensory attentiveness should occur over brief time periods, as subjects experience and focus their introspective attention on embodied iconic narrative representations (see Table 2). More broadly, we should be able to measure how culturally contextualized narratives and relationships, rituals, learning tasks, skilled artistic or craft production, and responses to scenarios involving culturally relevant power structures influence neural activity, vital rates, pupil dilation, and hormone levels within and between study groups defined by biological life history stages and culturally relevant identities.

## Conclusion

If the embodied cognition theoretical framework explains behavioral, central nervous system, and conceptual or representational phenomena better than strictly computational brain models—and certainly better than “disembodied” theories of mind (as argued by Clark, 2008 and Dove, 2011)—then paleoanthropological inquiry would benefit from embodied cognition research. Such interdisciplinary borrowing would facilitate investigating how unique-derived hominin brain anatomy and behavior patterns evolved, potentially helping to demystify the prehistoric emergence of language, symbolic representation, and the conscious human mind (Barton, 2012). In this article I have argued that ENC in the hominin lineage has involved a distinctive, semiotically structured and structuring interface between the body and the extrasomatic environment. This interface is constituted by narratives that are at once embodied and semiotically constructed, at once cognitive adaptations and embodied niche components. As we expand our perspective to view embodied cognition and interaction with the environment as both phenotype and niche, the ENC hypothesis can help to clarify the long-term evolutionary process through which human biology, semiotically structured worlds, and embodied experiences have emerged.

### Conflict of interest statement

The author declares that the research was conducted in the absence of any commercial or financial relationships that could be construed as a potential conflict of interest.

## Footnotes

^1^Maternal body mass may be estimated, even from fragmentary weight-bearing bones (especially the pelvis, femur, and tibia), based on extant human and ape population correlations between skeletal element morphology/allometric scaling and adult body mass (McHenry, 1992; Ruff, 2010; DeSilva, 2011). Neonate body mass estimates may be independently derived from endocranial volume measurements, elegantly based on constant anthropoid proportions between maternal and neonate brain mass, coupled with great ape and human constant proportions between brain and body mass (DeSilva and Lesnik, 2008; DeSilva, 2011; Tůma and Brůžek, 2013).
